# Chemical Characteristics and Antioxidant Properties of Crude Water Soluble Polysaccharides from Four Common Edible Mushrooms

**DOI:** 10.3390/molecules17044373

**Published:** 2012-04-11

**Authors:** Jin-Zhe He, Qiao-Mei Ru, Dan-Dan Dong, Pei-Long Sun

**Affiliations:** College of Biological and Environmental Engineering, Zhejiang University of Technology, Hangzhou 310032, China; Email: hejzgd@163.com (J.-Z.H.); ruqiaomei@zjut.edu.cn (Q.-M.R.); ranranbudong@yahoo.com.cn (D.-D.D.)

**Keywords:** edible mushroom, polysaccharide, chemical characteristics, antioxidant property

## Abstract

Four crude water soluble polysaccharides, CABP, CAAP, CFVP and CLDP, were isolated from common edible mushrooms, including *Agaricus bisporus*, *Auricularia auricula*, *Flammulina velutipes* and *Lentinus edodes*, and their chemical characteristics and antioxidant properties were determined. Fourier Transform-infrared analysis showed that the four crude polysaccharides were all composed of β-glycoside linkages. The major monosaccharide compositions were D-galactose, D-glucose and D-mannose for CABP, CAAP and CLDP, while CFVP was found to consist of L-arabinose, D-galactose, D-glucose and D-mannose. The main molecular weight distributions of CABP and the other three polysaccharides were <5.1 × 10^4^ Da and >66.0 × 10^4^ Da, respectively. Antioxidant properties of the four polysaccharides were evaluated in *in vitro* systems and CABP showed the best antioxidant properties. The studied mushroom species could potentially be used in part of well-balanced diets and as a source of antioxidant compounds.

## Abbreviations:

CABP: crude polysaccharide of *Agaricus bisporus*
CAAP: crude polysaccharide of * Auricularia auricular*
CFVP: crude polysaccharide of *Flammulina velutipes*
CLDP: crude polysaccharide of *Lentinus edodes*
L-Ara: L-arabinose
Ery: erythrose
L-Fuc: L-fucose
D-Gal: D-galactose
D-Glu: D-glucose
D-Man: D-mannose
L-Rha: L-rhamnose
D-Rib: D-ribose
D-Xyl: D-xylose

## 1. Introduction

Reactive oxygen species production is a common reaction and an essential biological process during normal cell metabolism. However, excess of reactive oxygen species can result in many diseases and accelerates ageing [1,2]. Therefore, it is essential to develop and utilize effective antioxidants that can scavenge free radicals in the human bodies. Due to the fact that most of antioxidants used are synthetic and have been suspected of being responsible for liver damage and carcinogenesis [3], it is essential to develop and utilize effective natural antioxidants.

Edible mushrooms have been valuable components of the human diet for thousands of years. In most countries, there is a well-established consumer acceptance, not only for their unique flavor and texture, but also for their chemical and nutritional properties [4,5]. Nowadays, edible mushrooms have become an attractive functional food mainly due to their chemical composition of polysaccharides. Recently, polysaccharides from *Agaricus bisporus* and *Agaricus brasiliensis* [6], wild edible mushrooms of *Armillaria mellea*, *Calocybe gambosa*, *Clitocybe odora* and *Coprinus comatus* [7], *Auricularia polytricha* [8], *Lentinula edodes *[9], *Morchella esculenta* [10] and *Pholiota adiposa* [11] have been obtained and their antioxidant properties have been investigated.

The objective of the present study was to evaluate and compare the chemical characteristics and antioxidant properties of crude water soluble polysaccharides from four species of common edible mushrooms: *Auricularia auricula*, *Agaricus bisporus*, *Flammulina velutipes* and *Lentinus edodes*. The four crude polysaccharides were termed as CABP, CAAP, CFVP and CLDP for *A. bisporus*, *A. auricular*, *F. velutipes* and *L. edode*s, respectively. Chemical characteristics studied were polysaccharide profile, monosaccharide composition and molecular weight distribution. Furthermore, the *in vitro* antioxidant potentials were evaluated by scavenging abilities of hydroxyl, superoxide anion and 1,1-dihpenyl-2-picrylhydrazyl (DPPH) radicals, and reducing power ability. This study is the first to compare these crude polysaccharides from four common edible mushrooms including chemical characteristics and antioxidant properties.

## 2. Results and Discussion

### 2.1. FT-IR Spectra

**Figure 1:**
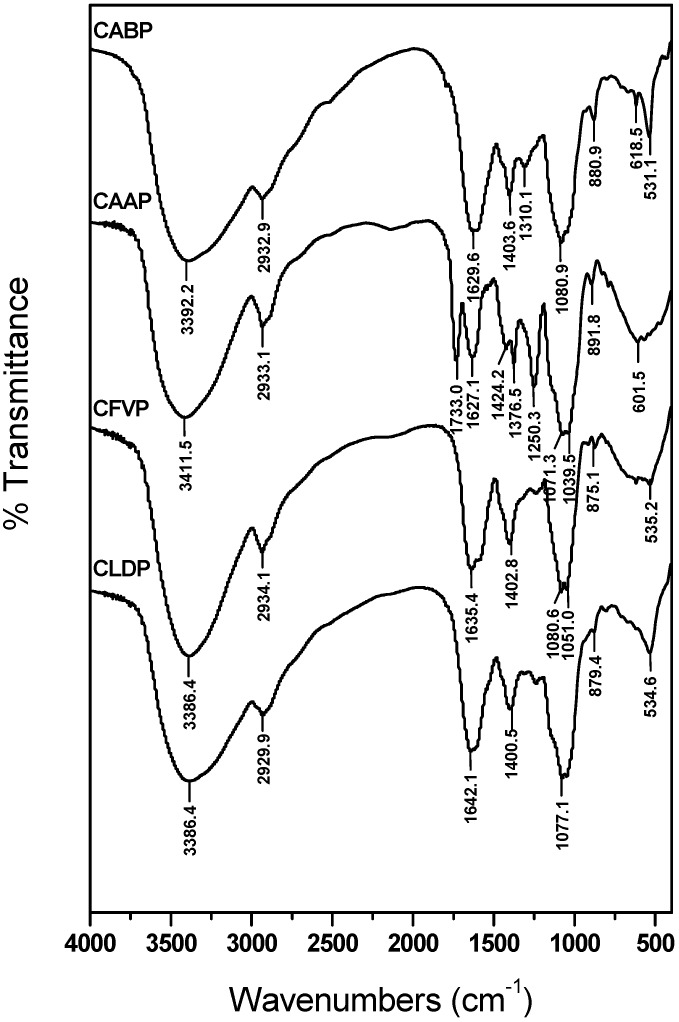
FT-IR spectra of the polysaccharides isolated from four species of edible mushrooms.

The FT-IR data showed that the four polysaccharides had typical carbohydrate patterns (Figure 1). The same characteristic strong broad bands near 3,400 cm^−^^1^ (CABP: 3,392.2 cm^−1^, CAAP: 3,411.5 cm^−1^, CFVP: 3,386.4 cm^−1^, CLDP: 3,386.4 cm^−^^1^) indicated the presence of OH stretching in hydrogen bonds which was indicative of strong inter- and intramolecular interaction of the polysaccharide chains. The weak band at around 2,932 cm^−1^ (CABP: 2,932.9 cm^−^^1^, CAAP: 2,933.1 cm^−^^1^, CFVP: 2,934.1 cm^−^^1^, CLDP: 2,929.9 cm^−^^1^) was attributed to the C–H stretching and bending vibrations. Carboxylate groups (C=O) showed two bands: an asymmetrical stretching band around 1,650 cm^−^^1^ (CABP: 1,629.6 cm^−1^, CAAP: 1,627.1 cm^−1^, CFVP: 1,635.4 cm^−1^, CLDP: 1,642.1 cm^−1^) and a weaker symmetric stretching band near 1,400 cm^−1^ (CABP: 1,403.6 cm^−1^, CAAP: 1,424.2 cm^−1^, CFVP: 1,402.8 cm^−1^, CLDP: 1,400.5 cm^−1^). The characteristic band nearby 1,080 cm^−^^1^ (CABP: 1,080.9 cm^−1^, CAAP: 1,071.3 cm^−1^, CFVP: 1,080.6 cm^−1^, CLDP: 1,077.1 cm^−1^) suggested a pyranose form of the glucosyl residue. The characteristic peak at around 880 cm^−1^ (CABP: 880.9 cm^−1^, CAAP: 891.8 cm^−1^, CFVP: 875.1 cm^−1^, CLDP: 879.4 cm^−1^) was typical for β-conﬁguration of the sugar units.

On the other hand, CAAP had more FT-IR features compared with the other three polysaccharides, CLDP, CABP and CFVP. The relatively strong absorption peak at 1,733.0 cm^−1^ corresponded to a carboxylic ester band, and the characteristic absorption at 1,376.5 cm^−^^1^ was assigned to C–H in-plane bending vibration. Moreover, the signal at 1,250.3 cm^−^^1^ was due to C–C band stretching vibrations from a ketone sugar, which were only present in CAAP.

### 2.2. Monosaccharide Composition

The monosaccharide composition results of the polysaccharides from four edible mushroom species are summarized in Table 1. From GC, the three polysaccharides, CABP, CAAP and CLDP, were found to mainly contain D-mannose, D-galactose and D-glucose. The monosaccharide contents were different for the three polysaccharides. For CABP, the contents of D-mannose, D-galactose and D-glucose were 36.68%, 20.70% and 34.85%, respectively. CAAP contained, in addition to D-glucose (64.40%), considerable proportions of D-mannose (18.23%) and D-galactose (7.84%). The most important monosaccharides in CLDP were D-glucose (59.90%), D-galactose (17.80%) and D-mannose (15.34%). CFVP was composed of four main monosaccharides. L-Arabinose was observed in CFVP and the content of L-arabinose (36.77%) was higher than the other three monosaccharides. The other main monosaccharides contained in CFVP were also D-glucose (34.18%), D-mannose (14.54%) and D-galactose (9.43%).

**Table 1:** Monosaccharide composition of the polysaccharidesisolated from four species of edible mushrooms.

Polysaccharides	Monosaccharide Content (%)								
	L-Ara	Ery	L-Fuc	D-Gal	D-Glu	D-Man	L-Rha	D-Rib	D-Xyl
CABP	1.26	0.70	1.10	20.70	34.85	36.68	0.62	1.00	3.10
CAAP	1.15	1.11	0.92	7.84	64.40	18.23	2.69	0.92	2.74
CFVP	36.77	0.57	2.10	9.43	34.18	14.54	1.01	1.10	0.30
CLDP	1.92	0.48	2.58	17.80	59.90	15.34	– ^a^	1.26	0.71

^a^ Undetectable.

### 2.3. Molecular Weight Distribution

Gel permeation chromatography (GPC) is an effective method for polysaccharide molecular weight determination. The calibration curve for molecular weight determination was made using a series of β-glucan standards: lg*M_w_* = −0.376V_t_+ 11.5 (R^2^ = 0.995), where *M_w_* is molecular weight; V_t_ is retention time. Based on the calibration curve made with a set of β-glucan series standards, Empower software was used for the calculation of molecular weights [12]. The main molecular weight distributions of four polysaccharides were shown in Table 2. CLDP showed a single molecular weight distribution, and its molecular weight distribution was 78.0 × 10^4^ Da. The other three polysaccharides showed two or more molecular weight distributions. CABP and CFVP had two different molecular weight distributions. For CABP, the main molecular weight distributions were in 5.09 × 10^4^ Da and minor in 2.17 × 10^4^ Da. For CFVP, the main molecular weight distributions were in 68.1 × 10^4^ Da and 65.5 × 10^4^ Da. CAAP had three different molecular weight distributions, primarily in 66.3 × 10^4^ Da, and secondarily in 1.32 × 10^4^ Da and 0.92 × 10^4^ Da, which was consistent with previous report that CAAP was a heteropolysaccharide [13].

**Table 2:** Molecular weight distribution of the polysaccharides isolated from four species of edible mushrooms.

Polysaccharides	Molecular weight distribution (×10^4^ Da)			
	Main distribution		Minor distribution	
CABP	5.09		2.17	
CAAP	66.3		1.32	0.92
CFVP	68.1	65.5		
CLDP	78.0			

### 2.4. Antioxidant Properties

For measuring antioxidant activities, different methods have been used corresponding to different levels of antioxidant action. In this experiment, the *in vitro* antioxidant capacities of four polysaccharides were evaluated using different biochemical methods of hydroxyl, superoxide anion and DPPH radical scavenging assay, and reducing power analysis.

#### 2.4.1. Scavenging Ability on Hydroxyl Radicals

**Figure 2:**
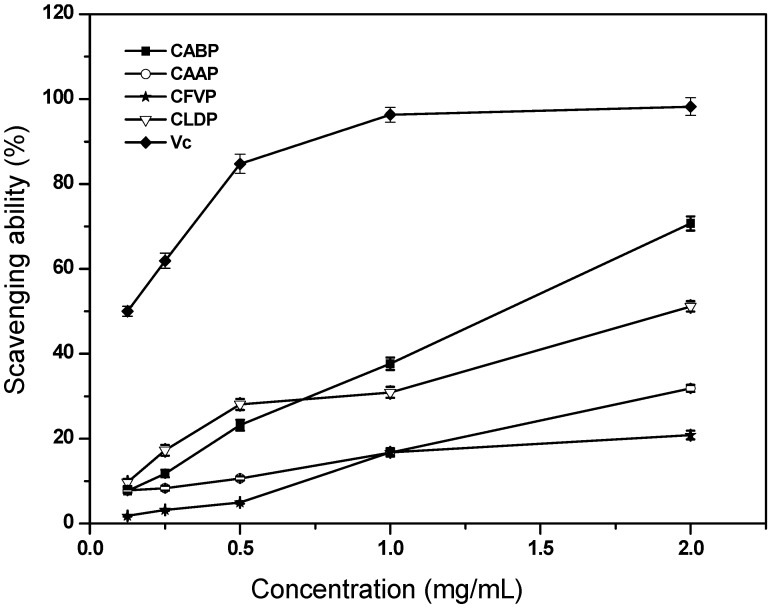
Scavenging ability of the polysaccharides isolated from four species of edible mushrooms on hydroxyl radicals.

Removing hydroxyl radicals is important for the protection of living systems because hydroxyl radicals are considered to be a highly potent oxidant, which can react with most biomacromolecules functioning in living cells and induce severe damage to the adjacent biomolecules. Scavenging abilities of the four polysaccharides isolated from four species of edible mushrooms on hydroxyl radicals were depicted in Figure 2. In this work, the scavenging abilities of CABP and CAAP on hydroxyl radicals were concentration-dependent, however, the scavenging abilities on hydroxyl radicals of CFVP and CLDP were not very concentration dependent, at the tested concentration range of 0.125–2.0 mg/mL. At 1.0 mg/mL, the scavenging abilities of CABP, CAAP, CFVP and CLDP on hydroxyl radicals were 37.63%, 16.68%, 16.74% and 30.89%, respectively. At 2.0 mg/mL, the scavenging abilities of CABP, CAAP, CFVP and CLDP were 70.68%, 31.84%, 20.84% and 51.16%, respectively. Moreover, at 2.0 mg/mL, CABP was found to have a higher scavenging activity on hydroxyl radicals than CAAP, CFVP and CLDP (*p* < 0.05). Effectiveness in antioxidant properties is inversely correlated with EC_50_ values. It is important to note the effectiveness of polysaccharides in reacting with free radicals under different conditions as a lower EC_50_ value corresponds to higher antioxidant activity of the edible mushroom’s polysaccharides. The EC_50_ values of scavenging ability on hydroxyl radicals for CABP and CLDP were 1.05 mg/mL and 1.90 mg/mL, respectively (Table 3). However, the EC_50_ values of scavenging ability on hydroxyl radicals were both >2 mg/mL for CAAP and CFVP (Table 3). The present results proved that CABP was a good scavenger for hydroxyl radicals. The polysaccharides minimized the concentration of Fe^2+^ in the Fenton reaction, and the scavenging abilities of the polysaccharides might be due to the active hydrogen donating ability of hydroxyl substitutions of polysaccharides. Therefore, CABP had a better potency to donate hydrogen to reactive hydroxyl radicals than CAAP, CFVP and CLDP. However, the scavenging abilities of the four polysaccharides on hydroxyl radicals were all relatively lower than that of Vc at the same concentrations. The EC_50_ values for hydroxyl radical-scavenging ability of CABP and CLDP were lower than that of the polysaccharide from another common edible mushroom, *Auricularia polytricha*, which was about 2.4 mg/mL [8]. However, the value of EC_50_ of the intracellular polysaccharide from one precious edible mushroom, *Pholiota adipose*, was 0.042 mg/mL [11], remarkably much lower than that of the four crude water soluble polysaccharides in this experiment.

**Table 3:** EC50 values of the polysaccharides isolated from four species of edible mushrooms.

	EC_50_ value (mg crude polysaccharide/mL)			
	CABP	CAAP	CFVP	CLDP
Scavenging ability on hydroxyl radicals	1.05	>2	>2	1.90
Scavenging ability on superoxide radicals	1.17	>2	>2	>2
Scavenging ability on DPPH radicals	0.55	1.62	>2	0.53
Reducing power	1.35	>2	>2	>2

#### 2.4.2. Scavenging Ability on Superoxide Anion Radicals

**Figure 3:**
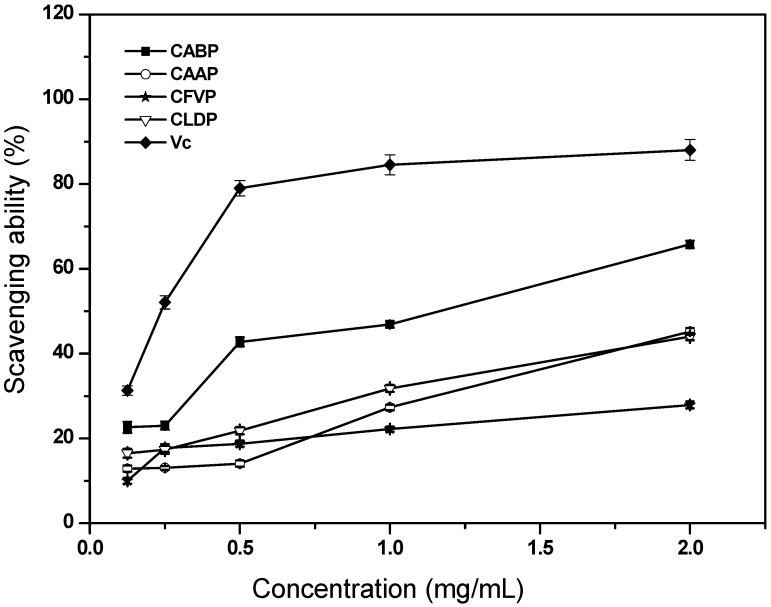
Scavenging ability of the polysaccharides isolated from four species of edible mushrooms on superoxide anion radicals.

Superoxide anions are a precursor of active free radicals react with biological macromolecules and induce tissue damage and play important roles in the formation of other reactive oxygen species such as hydrogen peroxide, hydroxyl radicals, and singlet oxygen, which induce oxidative damage in lipids, proteins and DNA [14]. Therefore, the superoxide radical-scavenging ability is of great importance to its potential antioxidant activity. Figure 3 reveals the scavenging abilities of the four crude polysaccharides on superoxide anion radicals. An increase of the scavenging activity on hydroxyl anion radicals was observed with the increasing tested concentration at the concentration range (0.125–2.0 mg/mL) of the polysaccharides, but didn’t exhibit a visible concentration-dependent manner. The scavenging effects of CABP, CAAP, CFVP and CLDP on superoxide anion radicals were 46.84%, 27.29%, 22.2% and 31.77%, respectively, at 1.0 mg/mL. The scavenging effects of CABP, CAAP, CFVP and CLDP on superoxide anion radicals were 65.78%, 45.21%, 27.9% and 43.99% at 2.0 mg/mL, respectively. CABP revealed a better antioxidant activity because the EC_50_ values of scavenging ability on superoxide anion radicals for CABP and the other three polysaccharides were 1.17 mg/mL and >2 mg/mL, respectively (Table 3). These results suggested that CABP was an effective scavenger for superoxide anion radicals, and it might be advantageous for preventing injury induced by superoxide radicals in pathological conditions. However, the four studied polysaccharides showed lower scavenging activities on superoxide radicals than Vc. The EC_50_ value of scavenging ability on superoxide anion radicals for the intracellular polysaccharide from *P. adiposa* was 0.0196 mg/mL [11], indicating much stronger superoxide anion radical-scavenging ability than the four crude water soluble polysaccharides in this experiment.

#### 2.4.3. Scavenging Ability on DPPH Radicals

**Figure 4:**
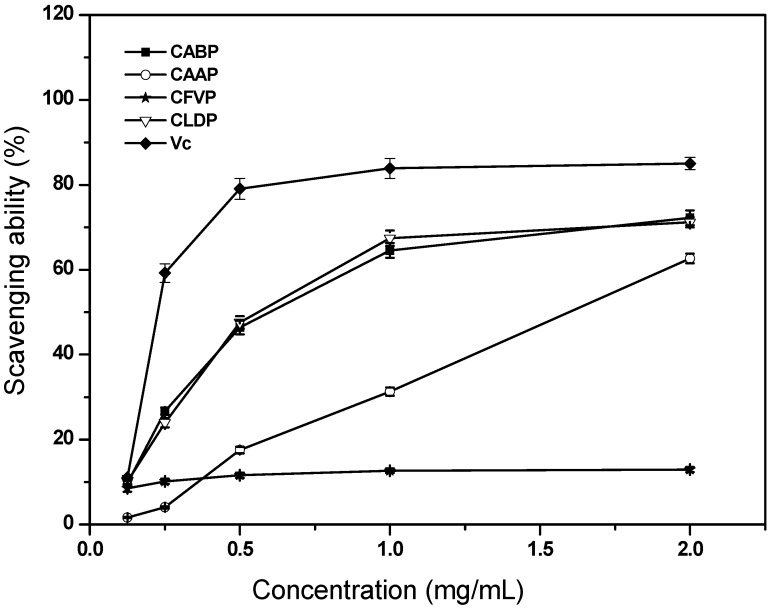
Scavenging ability of the polysaccharides isolated from four species of edible mushrooms on DPPH radicals.

The model of scavenging the stable DPPH radicals has been widely accepted as a tool to evaluate the free radical-scavenging activities of materials [15]. Figure 4 describes the scavenging abilities of different polysaccharides from four species of edible mushrooms on DPPH radicals. At all concentrations tested, CABP, CAAP and CLDP exhibited a dose-dependent DPPH radical-scavenging activity. Nevertheless, for CFVP, the scavenging ability on DPPH radicals slightly increased with increasing sample concentration. In the lower concentration ranged from 0.125 to 1.0 mg/mL, the scavenging abilities on DPPH radicals for CABP and CLDP were increased with increasing sample concentration, which were 64.56% and 67.39%, respectively, at 1.0 mg/mL. However, at 2.0 mg/mL, the scavenging abilities on DPPH radicals for CABP and CLDP were 72.24% and 71.16%, respectively, indicating no significantly increasing ability (*p* < 0.05). With regard to scavenging ability on DPPH radicals, CABP and CLDP showed very good scavenging abilities as evidenced by their particularly low EC_50_ values (≈0.5 mg/mL) (Table 3). For CAAP and CFVP, the EC_50_ values of scavenging ability on DPPH radicals were 1.62 and >2.0 mg/mL, respectively (Table 3). These results suggested that CABP and CLDP had higher scavenging abilities than CAAP and CFVP on DPPH radicals. It might be that CABP and CLDP could be better advantageous than CAAP and CFVP for reacting with DPPH radicals to convert them to more stable products and thereby terminate radical chain reactions. However, the scavenging abilities on DPPH radicals of the four polysaccharides were lower than that of Vc. The EC_50_ value of the polysaccharide from *A. bisporus* for DPPH radical-scavenging ability was 2 mg/mL [6], much higher than that of CABP. Moreover, the EC_50_ values of the polysaccharides from wild edible mushrooms of *Armillaria mellea*, *Calocybe gambosa*, *Clitocybe odora* and *Coprinus comatus* for DPPH radical-scavenging ability were 3.95 mg/mL, 7.08 mg/mL, 3.56 mg/mL and 7.31 mg/mL, respectively [7], which were remarkably much higher than that of CABP, CAAP and CLDP. It was worthy of noting that the EC_50_ value for DPPH radical-scavenging ability of the polysaccharide from another edible mushroom, *Agaricus brasiliensis*, was 0.27 mg/mL [6], much lower than that of the four polysaccharides in this experiment.

#### 2.4.4. Reducing Power

**Figure 5:**
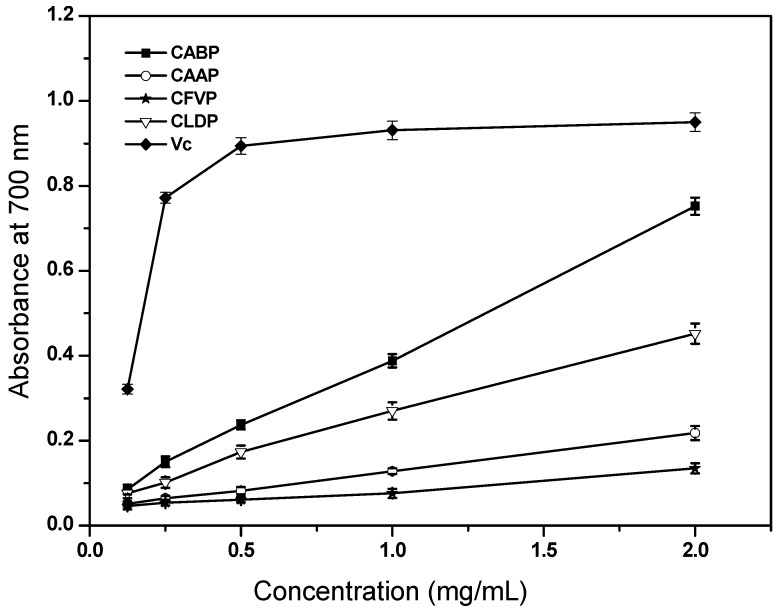
Reducing power of the polysaccharides isolated from four species of edible mushrooms.

The reducing capacity of a compound may serve as a signiﬁcant indicator of its potential antioxidant activity [16]. In this assay, the concentration-dependent proﬁle of reducing power was obvious for all the tested polysaccharides (Figure 5). At 1.0 mg/mL, the reducing powers were 0.388, 0.128, 0.076 and 0.270 for CABP, CAAP, CFVP and CLDP, respectively. At 2.0 mg/mL, the reducing powers were 0.752, 0.218, 0.135 and 0.452 for CABP, CAAP, CFVP and CLDP, respectively. With regard to reducing power, CABP showed the best antioxidant property because of its lowest EC_50_ value (1.35 mg/mL), indicating that CABP was good effective as an antioxidant (Table 3). High reducing power of CABP suggested a high potential in hydrogen-donating ability which could react with free radicals to convert them to more stable products and thereby terminate radical chain reactions [17]. CABP showed a obviously higher ability than that of the other three polysaccharides, but less than that of Vc. Kozarski *et al.* [6] and Vaz *et al.* [7] reported that the EC_50_ values for reducing power of the polysaccharides from *A. brasiliensis*, *Calocybe gambosa* and *Coprinus comatus* were 3.13 mg/mL, 2.38 mg/mL and 4.67 mg/mL, respectively, which were higher than that of CABP. Furthermore, for reducing power of the polysaccharide from *A. bisporus*, EC_50_ value was found of 14.83 mg/mL [6], remarkably higher than that of CABP studied in our experiment. However, the polysaccharides of *A. mellea* and *C. odora* showed the lower EC_50_ values for reducing power (0.98 mg/mL and 0.94 mg/mL, respectively) than CABP [7].

In general, it is interesting and important to elucidate the relation among chemical structures and chain conformations of polysaccharides and their biological activities [18]. The antioxidant activities of the polysaccharides might be related to monosaccharide component, molecular size, structure and conformation [19]. Tsiapali *et al.* [20] stated that the antioxidant abilities were partially related to monosaccharide composition. Moreover, Lo *et al.* [9] reported that the monosaccharide composition and the type of glycosyl linkage modulated the antioxidant properties of the polysaccharides. The antioxidant properties of the polysaccharides were dependent on the ratio of different monosaccharides in the composition. Among the monosaccharides, rhamnose was the most signiﬁcant determinant factor associated with antioxidant properties. The glycosyl linkage of the monosaccharides also affected the antioxidation characteristics of the polysaccharides. Speciﬁcally, the arabinose 1→4 and mannose 1→2 linkages of the side-chain were signiﬁcantly related to the reducing power, whereas the glucose 1→6 linkage and arabinose 1→4 linkage were related to the scavenging on DPPH radicals. In addition, a correlation was found between EC_50_ value of the reducing power ability and the amount of total glucans content in polysaccharides [6]. Furthermore, Luo *et al.* [18] figured out that the antioxidant activities of polysaccharides were supposed to relate to the structural characteristics of the polysaccharides, including molecular weight, monosaccharide composition and conﬁguration. Polysaccharides owned smaller molecular weight exhibited stronger antioxidant activities. Polysaccharides with β-conﬁguration in pyranose form sugars also exhibited stronger antioxidant activities. Besides, the high content of rhamnose was supposed to relate to the strong antioxidant effects.

In our experiment, the four crude polysaccharides showed different antioxidant activities. However, it is hard to clear the correlation between chemical characteristics and antioxidant properties of crude water soluble polysaccharides because using crude polysaccharides may cause many factors of chemical characteristics to influence their antioxidant properties. The objective of our study was to evaluate and compare the chemical characteristics and antioxidant properties of crude water soluble polysaccharides from four species of common edible mushrooms. Our results can give guidance for well-balanced diets in our daily life and a source of antioxidant compounds. Meanwhile, the correlation between chemical characteristics and antioxidant properties for purified polysaccharide fractions is undergoing in our lab. 

## 3. Experimental

### 3.1. Materials and Chemicals

Fresh fruiting bodies of *A. bisporus*, *A. auricular*, *F. velutipes* and *L. edodes* were collected from Zhejiang Agriculture Research Institute, Longquan City, Jiangshan City and Qingyuan County in Zhejiang Province, China, respectively. The voucher specimens were deposited in the College of Biological and Environmental Engineering, Zhejiang University of Technology. Vitamin C (Vc) and standard monosaccharides (L-arabinose, erythrose, L-fucose, D-galactose, D-glucose, D-mannose, L-rhamnose, D-ribose and D-xylose) were purchased from Sigma Chemical Co. (St. Louis, MO, USA). Six β-glucan series standards were purchased from Putus Macromolecular Sci. & Tech. Ltd. (Wuhan, China). All other chemicals were analytical grade and purchased from Shanghai Boer Chemical Reagent Co., Ltd. (Shanghai, China).

### 3.2. Isolation of the Polysaccharides

Four fruiting bodies of edible mushrooms were dried at 70 °C in oven and ground to obtain a fine powder (50 mesh), then weighed (50 g each) and extracted with 20 volumes of distilled water at 100 °C for 2 h. The suspension was centrifuged (8,000 × *g* for 15 min), and the supernatant was concentrated under vacuum. The protein in the product of condensation was deproteinized using the Sevag reagent [21]. After removal of the Sevag reagent, 4 volumes of anhydrous ethanol were added to this concentrate, and the mixture was stirred at room temperature for 20 min and then left at 4 °C overnight. The precipitate was collected by centrifugation (8,000 × *g* for 15 min), subsequently washed three times with anhydrous ethanol, acetone and ether, respectively, then dissolved in distilled water and dialyzed in dialysis bag (molecular weight cut-off, 1.2 × 10^4^ Da) against distilled water at room temperature for three successive days. The retained fraction was recovered, concentrated under vacuum and lyophilized to obtain four crude water soluble polysaccharides, which were termed as CABP, CAAP, CFVP and CLDP for *A. bisporus*, *A. auricular*, *F. velutipes* and *L. edode*s, respectively.

### 3.3. Fourier Transform-Infrared (FT-IR) Spectroscopy Analysis

The FT-IR spectra of polysaccharide extracts were recorded on a Thermo-Nicolet Model 6700 spectrophotometer (Thermo Scientific, Waltham, MA, USA). The spectrophotometer was equipped with DTGS TEC detector and OMNIC 7.3 software. Spectra were recorded in the 400–4,000 cm^−^^1^ range using KBr disc technique.

### 3.4. Monosaccharide Composition Analysis

For the monosaccharide composition analysis, four polysaccharides (5 mg each) were hydrolyzed with 2 M trifluoroacetic acid (5 mL) at 110 °C for 2 h. The hydrolyzate was repeatedly co-concentrated with methanol to dryness, subsequently reduced with NaBH_4_ for 3 h at room temperature and acetylated with acetic anhydride at 100 °C for 2 h, then concentrated with a little methylbenzene under reduced pressure. Finally, the acetylated monosaccharides were extracted by chloroform for gas chromatography (GC) analysis. The monosaccharide standards were acetylated in the same way. GC was performed on Agilent Technologies 7890A gas chromatograph with a DB-1701 capillary column (30 m × 0. 32 mm × 0.25 μm) and equipped with a flame ionization detector (FID). The nitrogen gas was used as the carrier gas at a ﬂow rate of 1 mL/min. The temperatures of injector and detector were set at 230 °C and 255 °C, respectively; the initial column temperature was increased from 150 °C at a rate of 10 °C/min to 222 °C and held for 16 min. These experiments were repeated twice.

### 3.5. Determination of Molecular Weight Distribution

Molecular weight distributions of polysaccharide samples from four edible mushroom species were evaluated and determined by high performance liquid chromatography (HPLC) with a Waters HPLC apparatus (Waters 515, Waters Co. Ltd., Milford, MA, USA) equipped with a 2424 refractive index detector (RID). Ultrahydrogel TM 500 (7.8 × 300 mm, Part NO. WAT11530) and Ultrahydrogel TM 1000 (7.8 × 300 mm, Part No. WAT11535) were used after connection in series and the temperature of the column was kept at 30 °C. The sample concentration was 0.8 mg/mL, and its injection volume was 10 μL. The eluent was 0.01 M NaNO_3_, and the ﬂow rate was 0.8 mL/min. The linear regression was calibrated with a set of β-glucan series standards of known molecular mass (*M_w_*: 1.2 × 10^4^ Da, 5.0 × 10^4^ Da, 8.0 × 10^4^ Da, 15.0 × 10^4^ Da, 27.0 × 10^4^ Da and 670 × 10^4^ Da), and then the retention times were plotted against the logarithms of their corresponding molecular weights. The retention times of the four polysaccharide samples were also plotted in the same graph, and the molecular weight distributions were determined.

### 3.6. Assay of Antioxidant Properties

#### 3.6.1. Scavenging Ability on Hydroxyl Radicals

Hydroxyl radical-scavenging activity was determined based on the method described by Smirnoff and Cumbes [22] with some modifications. The reaction mixture contained polysaccharide sample with different concentrations (0–2.0 mg/mL, 1 mL) was incubated with a solution containing orthophenanthroline (5 mM, 1 mL), phosphate buffer (7.5 mM, pH 7.4, 0.8 mL) and FeSO_4_ (7.5 mM, 0.5 mL). Finally, H_2_O_2_ (8.8 mM, 0.5 mL) was added, and the reaction mixture was then incubated at 37 °C for 1 h. The absorbance of the resulting solution was measured spectrophotometrically at 532 nm. The scavenging ability of hydroxyl radicals was calculated according to the equation: scavenging ability (%) = (1 − A_sample_/A_control_) × 100, where A_control_ is the absorbance of control without the polysaccharide sample, and A_sample_ is the absorbance in the presence of the polysaccharide sample. The EC_50_ value (mg/mL) is the effective concentration at which the hydroxyl radicals are scavenged by 50%. Vc was used for comparison because it is a standard antioxidant.

#### 3.6.2. Scavenging Ability on Superoxide Anion Radicals

The scavenging activity of superoxide anion radicals was assessed referring to the reference [23] with several modifications. A tube containing Tris-HCl buffer (50.0 mM, pH 8.2, 3 mL) and polysaccharide sample (0–2.0 mg/mL, 1 mL) was incubated in a water bath at 25 °C for 20 min, then pyrogallic acid (5.0 mM, 0.4 mL) at the same temperature was added and proceed at 25 °C. HCl solution (8.0 M, 0.1 mL) was used to terminate the reaction after 4 min. The absorbance of the mixture was measured at 320 nm. The scavenging ability of superoxide anion radicals was calculated using the following formula: scavenging ability (%) = (1 − A_sample_/A_control_) × 100, where A_control_ is the absorbance of control without the polysaccharide sample, and A_sample_ is the absorbance in the presence of the polysaccharide sample. The EC_50_ value (mg/mL) is the effective concentration at which the superoxide anion radicals are scavenged by 50%. For comparison, Vc was used as positive control.

#### 3.6.3. Scavenging Ability on 1,1-Diphenyl-2-picrylhydrazyl Radicals

The DPPH radical-scavenging activity was measured according to the method of Braca *et al.* [24]. Polysaccharide sample with different concentrations (0–2.0 mg/mL, 1 mL) was mixed with methanol solution (2 mL) containing DPPH radicals (0.2 mM). The mixture was shaken vigorously and incubated for 30 min in darkness at room temperature, and then absorbance at 517 nm was measured. The scavenging ability of DPPH radicals was calculated using the following formula: scavenging ability (%) = (1 − A_sample_/A_control_) × 100, where A_control _is the absorbance of control without the polysaccharide sample, and A_sample_ is the absorbance in the presence of the polysaccharide sample. The EC_50_ value (mg/mL) is the effective concentration at which the DPPH radicals are scavenged by 50%. Vc was used as standard antioxidant and positive control.

#### 3.6.4. Reducing Power Ability

The reducing power was determined referring to the method described in literature [25] with slight modifications. Polysaccharide sample with different concentrations (0–2.0 mg/mL, 1 mL) was mixed with phosphate buffer (0.2 M, pH 6.6, 2.5 mL) and K_3_Fe(CN)_6_ (1%, w/v, 2.5 mL). The mixture was incubated at 50 °C for 20 min, then trichloroacetic acid (10%, w/v, 2.5 mL) was added to the mixture, subsequently centrifuged at 3,000 × *g* for 10 min. An aliquot of supernatant (5 mL) was mixed with deionized water (4 mL) and FeCl_3_ (0.1%, w/v, 1 mL). After incubating at room temperature for 10 min, the absorbance of the mixture was measured at 700 nm. A higher absorbance in the reaction mixture indicates greater reducing power ability. Vc was used as positive control for comparison.

### 3.7. Statistical Analysis

For each polysaccharide, three samples were prepared for assays of every antioxidant attribute. All bioassay results were expressed as means ± standard deviation (SD) of three replications, and the one-way analysis of variance (ANOVA) was used for the statistical analysis.

## 4. Conclusions

In this study, four crude water soluble polysaccharides were isolated from four species of common edible mushrooms. Chemical characteristics, including polysaccharide profile, monosaccharide composition and molecular weight distribution of the four polysaccharides were determined. FT-IR analysis showed that the four crude polysaccharides were all composed of β-glycoside linkages. Monosaccharide composition results indicated the dominance of D-mannose, D-galactose and D-glucose in CABP, CAAP and CLDP with different contents of monosaccharide. CFVP was mainly composed of four monosaccharides, namely L-arabinose, D-mannose, D-galactose and D-glucose. The main molecular weight distributions of CABP and the other three polysaccharides were <5.1 × 10^4^ Da and >66.0 × 10^4^ Da, respectively. For antioxidant activities of the four polysaccharides, CABP was the best natural antioxidant. The water soluble polysaccharides of edible mushrooms acted as natural antioxidants could be most good sources of antioxidant foods and the consumption of edible mushrooms might give a certain level of health protection against oxidative damages.
